# Critically Ill Patients With Icu-Acquired Weakness Show Reduced Density of Myosin in Electron Microscope Early After Onset of Critical Illness

**DOI:** 10.1186/2197-425X-3-S1-A44

**Published:** 2015-10-01

**Authors:** T Rathke, T Wollersheim, M Krebs, M Schülke, S Weber-Carstens

**Affiliations:** Charité Universitätsmedizin Berlin, Campus Virchow & Campus Mitte, Berlin, Germany

## Introduction

Recently we showed increased expression of atrophy genes MuRF-1 and Atrogin-1 during early course of critical illness resulting in MyHC loss and finally the clinical presentation of ICU-acquired weakness. Appropriate studies of systematic electron microscope investigations from this early time in critical illness do not exist yet.

## Objectives

To investigate muscle ultrastructure with electron microscopy within the time course of critical illness.

## Methods

Controlled, prospective, monocentric observational study. We included 30 mechanically ventilated, critically ill patients (SOFA score ≥ 8 at 3 within 5 days after ICU admission). Two open muscle biopsies (*M. vastus lateralis*) were obtained at median day 5 and 16. Electron microscope studies were performed and counting of myosin filaments in cross section was done using Image J Software. Reference values were taken from Riley et al.. Muscle strength was assessed using Medical Research Council (MRC)-Scale when patients were alert later in ICU course. Non-parametric tests were performed. Ethic vote (Charité EA2/061/06).

## Results

We see a significant reduction of myosin filament to almost half the normal density already in the first biopsy: n/µm^2^(mean/SD): 577/193 vs.1054/71. There is no significant ongoing decrease of myosin filament later according to second biopsy - n/µm^2^(mean/SD):416/172. We found a significant correlation of myosin filament density and MRC-Scale for the first biopsy, yet not for the second biopsy.

**Figure 1:**
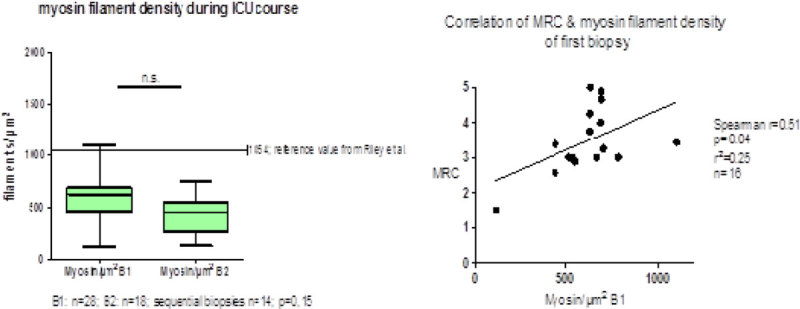
Figure 1

## Conclusions

Electron microscopy reveals a serious reduction of myosin filament density very rapidly after onset of critical illness. However, during course of critical illness the further reduction of myosin filament density is of minor degree. The early myosin filament loss determines development of ICU-acquired weakness later during ICU stay, when patients emerge from sedation.

## Grant Acknowledgment

Funded by DFG, KFO 192, WE 4386/1-2.
